# Supplementary material for: Altered Temporal Variability of Local and Large-Scale Resting-State Brain Functional Connectivity Patterns in Schizophrenia and Bipolar Disorder
Source: Front Psychiatry. 2020 May 12;11:422. doi: 10.3389/fpsyt.2020.00422 (PMC7235354; doi:10.3389/fpsyt.2020.00422)
Supplement: Supplementary file 2 [file Table_2.docx]

**Supplementary Table S2.** The detected significant between-group differences in temporal variabilities of regional functional connectivity for particular regions of interest.

| Region of interest | Main effect of group | Significant post-hoc pairwise comparisons*^a^* |
| --- | --- | --- |
| Right precentral gyrus | *F* = 6.625, *p* = 0.002 | Schizophrenia > healthy controls (*p* = 0.001) |
| Left superior frontal gyrus, medial orbital | *F* = 3.865, *p* = 0.023 | Schizophrenia < healthy controls (*p* = 0.032) |
| Left posterior cingulate gyrus | *F* = 3.482, *p* = 0.033 | Schizophrenia < bipolar disorder (*p* = 0.030) |
| Left hippocampus | *F* = 4.137, *p* = 0.018 | Schizophrenia > healthy controls (*p* = 0.029) |
| Left amygdala | *F* = 3.979, *p* = 0.020 | Schizophrenia > healthy controls (*p* = 0.017) |
| Right amygdala | *F* = 7.617, *p* = 0.001 | Schizophrenia > healthy controls (*p* = 0.0004) |
| Left postcentral gyrus | *F* = 3.277, *p* = 0.040 | Schizophrenia > healthy controls (*p* = 0.047) |
| Right postcentral gyrus | *F* = 3.547, *p* = 0.031 | Schizophrenia > healthy controls (*p* = 0.027) |
| Right inferior parietal lobule | *F* = 3.472, *p* = 0.033 | Schizophrenia > healthy controls (*p* = 0.028) |
| Right putamen | *F* = 3.483, *p* = 0.033 | Schizophrenia > healthy controls (*p* = 0.031) |
| Right pallidum | *F* = 4.246, *p* = 0.016 | Bipolar disorder > healthy controls (*p* = 0.015) |
| Left thalamus | *F* = 8.915, *p* = 0.0002 | Schizophrenia > healthy controls (*p* = 0.0002), bipolar disorder > healthy controls (*p* = 0.017) |
| Right thalamus | *F* = 7.386, *p* = 0.001 | Schizophrenia > healthy controls (*p* = 0.001) |

*^a^*The *p* values were Bonferroni-corrected for multiple tests within the analysis of covariance.
