# Supplementary material for: Altered Temporal Variability of Local and Large-Scale Resting-State Brain Functional Connectivity Patterns in Schizophrenia and Bipolar Disorder
Source: Front Psychiatry. 2020 May 12;11:422. doi: 10.3389/fpsyt.2020.00422 (PMC7235354; doi:10.3389/fpsyt.2020.00422)
Supplement: Supplementary file 3 [file Table_3.docx]

**Supplementary Table S3.** The detected significant between-group differences in temporal variabilities of intra-network and inter-network functional connectivity for particular networks or pairs of networks.

| Network/pair of networks | Main effect of group | Significant post-hoc pairwise comparisons*^a^* |
| --- | --- | --- |
| Visual | *F* = 3.359, *p* = 0.037 | Schizophrenia > healthy controls (*p* = 0.041) |
| Sensorimotor | *F* = 3.083, *p* = 0.048 | Schizophrenia > healthy controls (*p* = 0.049) |
| Sensorimotor-salience | *F* = 3.642, *p* = 0.028 | Schizophrenia > healthy controls (*p* = 0.023) |
| Sensorimotor-subcortical | *F* = 4.134, *p* = 0.018 | Schizophrenia > healthy controls (*p* = 0.014) |
| Thalamus-sensorimotor | *F* = 7.907, *p* = 0.001 | Schizophrenia > healthy controls (*p* = 0.0004), bipolar disorder > healthy controls (*p* = 0.036) |
| Thalamus-default-mode | *F* = 5.060, *p* = 0.007 | Schizophrenia > healthy controls (*p* = 0.005) |
| Subcortical-salience | *F* = 3.464, *p* = 0.033 | Schizophrenia > healthy controls (*p* = 0.028) |
| Subcortical-auditory | *F* = 5.529, *p* = 0.005 | Schizophrenia > healthy controls (*p* = 0.006) |

*^a^*The *p* values were Bonferroni-corrected for multiple tests within the analysis of covariance.
