# Supplementary material for: Altered Temporal Variability of Local and Large-Scale Resting-State Brain Functional Connectivity Patterns in Schizophrenia and Bipolar Disorder
Source: Front Psychiatry. 2020 May 12;11:422. doi: 10.3389/fpsyt.2020.00422 (PMC7235354; doi:10.3389/fpsyt.2020.00422)
Supplement: Supplementary file 4 [file Table_4.docx]

**Supplementary Table S4.** Demographic, clinical and head motion characteristics of each group in the subset, where the illness duration and antipsychotic dosage were matched between the schizophrenia and bipolar disorder groups.

|  | Schizophrenia (*n* = 48) | Bipolar disorder (*n* = 30) | Healthy controls (*n* = 56) | Group comparisons |
| --- | --- | --- | --- | --- |
|  | (Mean ± SD) | (Mean ± SD) | (Mean ± SD) |  |
| Age (years) | 24.396 ± 5.974 | 24.133 ± 3.748 | 23.196 ± 4.746 | *F* = 0.800, *p* = 0.451 |
| Sex (male/female) | 29/19 | 16/14 | 26/30 | *χ*^2^ = 2.032, *p* = 0.362 |
| Education (years) | 12.979 ± 2.047 | 13.167 ± 2.520 | 13.554 ± 1.972 | *F* = 0.977, *p* = 0.379 |
| Illness duration (months) | 23.604 ± 25.523 | 36.437 ± 31.144 | / | *t* = -1.983, *p* = 0.051 |
| Antipsychotics (taking/not taking) | 45/3 | 30/0 | / | *χ*^2^ = 0.626, *p* = 0.429 |
| Chlorpromazine equivalents (mg/day) | 183.547 ± 87.030 | 157.541 ± 121.699 | / | *t* = 1.099, *p* = 0.275 |
| SAPS scores | 18.298 ± 14.943 | / | / | / |
| SANS scores | 30.021 ± 27.662 | / | / | / |
| 17-item HAMD scores | / | 10.724 ± 9.277 | / | / |
| YMRS scores | / | 5.731 ± 9.396 | / | / |
| WAIS-I scores | 18.457 ± 4.318 | 19.267 ± 4.567 | 20.713 ± 5.042 | *F* = 2.965, *p* = 0.055 |
| WAIS-DS scores | 64.467 ± 14.890 | 68.733 ± 16.653 | 87.865 ± 13.686 | *F* = 33.500, *p* < 0.001*^a^* |
| Mean FD | 0.093 ± 0.038 | 0.090 ± 0.039 | 0.088 ± 0.032 | *F* = 0.258, *p* = 0.773 |

***^a^***The LSD post-hoc comparisons set at *p* < 0.05 showed that schizophrenia < healthy controls, and bipolar disorder < healthy controls, while there was no significant difference between the schizophrenia and bipolar disorder groups. SD, standard deviation; SAPS, Scale for Assessment of Positive Symptoms; SANS, Scale for Assessment of Negative Symptoms; HAMD, Hamilton Rating Scale for Depression; YMRS, Young Mania Rating Scale; WAIS-I, the Information subtest of the Wechsler Adult Intelligence Scale; WAIS-DS, the Digit Symbol subtest of the Wechsler Adult Intelligence Scale; FD, framewise-displacement.
