# Supplementary material for: Altered Temporal Variability of Local and Large-Scale Resting-State Brain Functional Connectivity Patterns in Schizophrenia and Bipolar Disorder
Source: Front Psychiatry. 2020 May 12;11:422. doi: 10.3389/fpsyt.2020.00422 (PMC7235354; doi:10.3389/fpsyt.2020.00422)
Supplement: Supplementary file 5 [file Table_5.docx]

**Supplementary Table S5.** The detected significant between-group differences in temporal variabilities of regional functional connectivity for particular regions of interest, when repeating the analyses with global signal regression.

| Region of interest | Main effect of group | Significant post-hoc pairwise comparisons*^a^* |
| --- | --- | --- |
| Left precentral gyrus | *F* = 3.107, *p* = 0.047 | Schizophrenia > healthy controls (*p* = 0.045) |
| Left hippocampus | *F* = 5.827, *p* = 0.004 | Schizophrenia > healthy controls (*p* = 0.016), Schizophrenia > bipolar disorder (*p* = 0.009) |
| Left parahippocampal gyrus | *F* = 3.210, *p* = 0.043 | Schizophrenia < bipolar disorder (*p* = 0.047) |
| Right parahippocampal gyrus | *F* = 3.611, *p* = 0.029 | Schizophrenia < bipolar disorder (*p* = 0.029) |
| Right putamen | *F* = 3.218, *p* = 0.042 | Schizophrenia > healthy controls (*p* = 0.036) |
| Right pallidum | *F* = 4.024, *p* = 0.020 | Bipolar disorder > healthy controls (*p* = 0.018) |
| Left thalamus | *F* = 6.493, *p* = 0.002 | Schizophrenia > healthy controls (*p* = 0.002), bipolar disorder > healthy controls (*p* = 0.047) |
| Right thalamus | *F* = 3.435, *p* = 0.034 | Schizophrenia > healthy controls (*p* = 0.029) |

*^a^*The *p* values were Bonferroni-corrected for multiple tests within the analysis of covariance.
