# Supplementary material for: Altered Temporal Variability of Local and Large-Scale Resting-State Brain Functional Connectivity Patterns in Schizophrenia and Bipolar Disorder
Source: Front Psychiatry. 2020 May 12;11:422. doi: 10.3389/fpsyt.2020.00422 (PMC7235354; doi:10.3389/fpsyt.2020.00422)
Supplement: Supplementary file 6 [file Table_6.docx]

**Supplementary Table S6.** The detected significant between-group differences in temporal variabilities of intra-network and inter-network functional connectivity for particular networks or pairs of networks, when repeating the analyses with global signal regression.

| Network/pair of networks | Main effect of group | Significant post-hoc pairwise comparisons*^a^* |
| --- | --- | --- |
| Sensorimotor | *F* = 3.425, *p* = 0.035 | Schizophrenia > healthy controls (*p* = 0.049) |
| Sensorimotor-subcortical | *F* = 3.894, *p* = 0.022 | Schizophrenia > healthy controls (*p* = 0.021) |
| Thalamus-sensorimotor | *F* = 7.439, *p* = 0.001 | Schizophrenia > healthy controls (*p* = 0.001), bipolar disorder > healthy controls (*p* = 0.021) |
| Thalamus-attention | *F* = 3.428, *p* = 0.035 | Schizophrenia > healthy controls (*p* = 0.029) |
| Subcortical-salience | *F* = 3.924, *p* = 0.022 | Schizophrenia > healthy controls (*p* = 0.027) |
| Subcortical-auditory | *F* = 3.967, *p* = 0.021 | Schizophrenia > healthy controls (*p* = 0.016) |

*^a^*The *p* values were Bonferroni-corrected for multiple tests within the analysis of covariance.
