# Supplementary material for: Altered Temporal Variability of Local and Large-Scale Resting-State Brain Functional Connectivity Patterns in Schizophrenia and Bipolar Disorder
Source: Front Psychiatry. 2020 May 12;11:422. doi: 10.3389/fpsyt.2020.00422 (PMC7235354; doi:10.3389/fpsyt.2020.00422)
Supplement: Supplementary file 7 [file Table_7.docx]

**Supplementary Table S7.** The detected significant between-group differences in temporal variabilities of regional, intra-network and inter-network functional connectivity, when repeating the analyses within a subset where the illness duration and antipsychotic dosage were matched between the schizophrenia and bipolar disorder groups.

| Region of interest/ network/pair of networks | Main effect of group | Significant post-hoc pairwise comparisons*^a^* |
| --- | --- | --- |
| Right precentral gyrus | *F* = 5.514, *p* = 0.002 | Schizophrenia > healthy controls (*p* = 0.004) |
| Right amygdala | *F* = 4.873, *p* = 0.009 | Schizophrenia > healthy controls (*p* = 0.009) |
| Right postcentral gyrus | *F* = 3.289, *p* = 0.040 | Schizophrenia > healthy controls (*p* = 0.035) |
| Right pallidum | *F* = 3.263, *p* = 0.042 | Bipolar disorder > healthy controls (*p* = 0.044) |
| Left thalamus | *F* = 6.003, *p* = 0.003 | Schizophrenia > healthy controls (*p* = 0.007), bipolar disorder > healthy controls (*p* = 0.022) |
| Right thalamus | *F* = 4.992, *p* = 0.008 | Schizophrenia > healthy controls (*p* = 0.007) |
| Thalamus-sensorimotor | *F* = 5.640, *p* = 0.004 | Schizophrenia > healthy controls (*p* = 0.006), bipolar disorder > healthy controls (*p* = 0.038) |
| Subcortical-auditory | *F* = 3.491, *p* = 0.033 | Schizophrenia > healthy controls (*p* = 0.048) |

*^a^*The *p* values were Bonferroni-corrected for multiple tests within the analysis of covariance.
